# Tumour acquisition method and molecular profiling success in advanced cholangiocarcinoma

**DOI:** 10.2340/ao.v65.45466

**Published:** 2026-05-07

**Authors:** Eleni Vrana, Alia Alothman, Luke Taylor, Joe Geraghty, Javaid Iqbal, Lucy Foster, Alicia-Marie Conway, Nadina Tinsley, Melissa Frizziero, Victoria Foy, Richard A Hubner, Mairéad G. McNamara

**Affiliations:** a The Christie NHS Foundation Trust, Manchester, UK; b University of Manchester, Faculty of Biology, Medicine and Health, Manchester, UK; c Manchester Royal Infirmary, Manchester, UK; d University Hospital of South Manchester, Manchester, UK; e Division of Cancer Sciences/Department of Medical Oncology, University of Manchester/The Christie NHS Foundation Trust, Manchester, UK

**Keywords:** Cholangiocarcinoma, molecular profiling, biopsy, targeted treatment

## Introduction

Cholangiocarcinoma (CCA) incidence is rising and it remains a highly lethal disease with a poor prognosis [1–3]. The recent addition of immune checkpoint inhibitors (ICIs) such as durvalumab (TOPAZ-1) [4] and pembrolizumab (KEYNOTE-966) [5] to the first-line cisplatin/gemcitabine [6] backbone has modestly improved median overall survival (mOS). However, with 3- and 5-year survival rates still below 10%, the quest for more efficient, personalised strategies are paramount [7, 8].

The shift towards precision oncology has been fuelled by genomic studies highlighting the genetic richness of CCA, particularly in the intrahepatic subtype (iCCA). While research indicates that around 40% of iCCA cases harbour alterations with potential therapeutic relevance [9], the proportion of patients for whom biomarker-directed therapy is currently a standard of care (SoC) indication remains more modest. In current practice, the most frequent and clinically relevant alterations include: *isocitrate dehydrogenase 1* (*IDH1*) mutations and *fibroblast growth factor receptor 2* (*FGFR2*) fusions/rearrangements [10]. In the second-line setting, targeted agents such as ivosidenib (*IDH1* inhibitor) and pemigatinib/futibatinib (*FGFR2* inhibitors) have established a new SoC for patients with these specific alterations, based on pivotal trials, i.e. ClarIDHy, FIGHT-202, FOENIX-CCA2, respectively [11–13]. Beyond these, the therapeutic landscape includes *BRAF serine/threonine kinase* mutations, *neurotrophic tyrosine receptor kinase* (*NTRK*) fusions, *erb-b2 receptor tyrosine kinase 2 (ERBB2)* amplifications, alongside alterations in the homologous recombination repair (HRR) machinery, such as *BRCA1/2*. In addition, while microsatellite instability/mismatch repair deficiency (*MSI-H/dMMR*) status is identified in only a small subset of patients, it remains a critical biomarker predictive of response to ICIs. The increasing diversity of these targets makes comprehensive molecular profiling an essential component of modern treatment planning.

## Tissue acquisition: the foundation of precision

This retrospective observational study included 154 consecutive patients with cytological or histological diagnosis of advanced CCA (baseline patient characteristics in Table 1), who were referred for consideration of systemic therapy to a tertiary referral centre between 2022 and 2024. The primary objective was to assess the molecular testing success rate based on the tissue acquisition method. Achieving adequate tumour cellularity, previously defined as ≥ 20% [14], and high-quality tissue is essential for successful Next Generation Sequencing and genomic analysis. To facilitate molecular analysis, all cytological specimens were processed using the cell block technique, converting aspirates and brushings into paraffin-embedded blocks.

**Figure 1:**
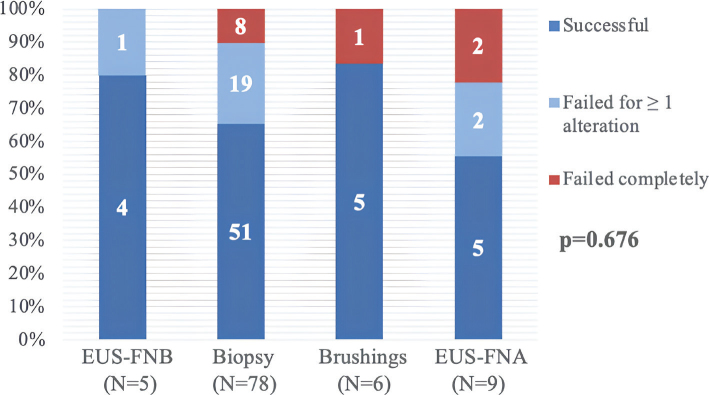
Tissue acquisition method success rate. X axis: tissue acquisition method, Y axis: molecular success rate. The molecular success rates were compared using the Fisher’s exact test. A p value < 0.05 was considered statistically significant. EUS-FNA: endoscopic ultrasound-fine needle aspiration; EUS-FNB: endoscopic ultrasound-fine needle biopsy.

- Molecular profiling was requested for 98/154 patients (64%) and it was performed using a multigene panel in 19/98 (19%) (FoundationOne^®^CDx that assesses 324 genes) and by National Health Service (NHS) Genomics (assessing *FGFR2, IDH1, MSI, NTRK*) in 79/98 (81%). Tumour cellularity was provided in 75 (49%) pathology reports out of the total 154.
- The main reason molecular testing was not requested (56/154 [46%]) were in cases where patient management decision was for best supportive care (BSC) [41/56 (73%)]. For the remaining 15/56 (27%) samples with no molecular testing request: 9 (16%) were cytology samples with no reported tumour cellularity, therefore deemed to result in an unsuccessful analysis; 6 (11%) were tissue samples with no reported tumour cellularity in 4 (7%) of them. For the 2 (4%) tissue samples with provided tumour cellularity molecular profiling was inadvertently omitted.
- Molecular profiling was successful for at least one targetable alteration in 83/98 (85%) of the requested samples, with availability of all NHS alteration results in 65/98 (67%), which is comparable to previously published cohorts in biliary tract cancer [15] (Table 1, Supplemental Table 1 and Supplemental Figure 1).
- Endoscopic ultrasound fine needle biopsy (EUS-FNB) and surgical/percutaneous biopsies demonstrated superior success rates compared to cytology-based approaches like EUS-fine needle aspiration (EUS-FNA) and biliary brushings, although this did not reach statistical significance, likely due to small sample size in the EUS-FNB group (Figure 1).
- The higher yield from EUS-FNB is consistent with data from other gastrointestinal malignancies, where core biopsy needles provide better histological preservation and higher nucleic acid yield than FNA, facilitating downstream molecular analysis [16].
- The high failure rate in the cohort [15/98 (15%) total failure (no results possible), 18/98 (18%) partial failure (inadequate for detection of some alterations)] highlights the need for careful tissue triage, involving pathologists selecting the most representative block and area enriched in neoplastic cells [17]. Interestingly, in our study, *IDH1* mutation analysis (a DNA-based test) failed most frequently [14 (14%) out of the 18 samples with partial failure, Supplemental Figure 2], underscoring the complexities even with relatively stable DNA targets.

**Table 1:** Baseline patient/sample characteristics and therapeutic management.

Variable	Number of patients (*N* = 154)
Age	70 years (60.2–75.8)
Gender	
Male	81 (53%)
Female	73 (47%)
ECOG PS	
0	27 (18%)
1	65 (42%)
2	36 (23%)
3	24 (16%)
4	2 (1%)
Primary	
iCCA	97 (63%)
pCCA	27 (18%)
dCCA	30 (19%)
Stage IV	98 (64%)
Tissue acquisition method	
Surgical/Percutaneous biopsy	104 (68%)
EUS-FNB	12 (8%)
EUS-FNA	11 (7%)
Brushings	27 (17%)
Acquired tissue	
Liver	94 (61%)
Bile duct	47 (30%)
LN	4 (3%)
Duodenum	1 (1%)
Omentum/peritoneum	5 (3%)
Bone	3 (2%)
Molecular profiling requests	98 (64%)
Molecular profiling success	87/98 (89%)
Total success (report for all relevant alterations)	65/98 (67%)
Failure for ≥ 1 targetable alteration (partial failure)	22/98 (22%)
Complete failure	11/98 (11%)
Molecular assay	
FoundationOne^®^CDx (assessing 324 genes)	19/98 (19%)
NHS genomics (assessing FGFR2, IDH1, MSI, NTRK)	79/98 (81%)
Tumour cellularity provided on report	75 (49%)
Adequacy of tumour cellularity	66/75 (88%)
Repeated analysis from a different tissue block in patients with partial or complete primary analysis failure	6/33 (18%)
Successful	5/6 (83%)
Liquid biopsy prior to 2^nd^ line in patients with partial or complete primary analysis failure	2/33 (6%)
No molecular profiling requests	56 (46%)
Reason for not requesting molecular profiling:	
BSC	41/56 (73%)
Absence of tumour cellularity report	13/56 (23%)
Inadvertent omission (tissue samples)	2/56 (4%)
Management throughout the whole course of the disease*	
SACT alone	94 (61%)
RT + Chemotherapy	2 (1%)
RT	1 (1%)
BSC	57 (37%)
Details of SACT	96 (62%)
Chemotherapy alone	67 (43%)
Chemotherapy + IO	20 (13%)
Chemotherapy + IO + targeted therapy**	2 (1%)
Chemotherapy + targeted therapy**	7 (5%)
Targeted treatment	9 (6%)
Ivosidenib	6 (4%)
	[4 (3%) in 2L & 2 (1%) in 3L]
	3 (2%)
Pemigatinib	[all in 2L]

Values are *n* (%), median with IQR. BSC: best supportive care; dCCA: distal cholangiocarcinoma; ECOG PS: Eastern Oncology Cooperative Group performance status; EUS-FNA: endoscopic ultrasound fine needle aspiration; EUS-FNB: endoscopic ultrasound fine needle biopsy; iCCA: intrahepatic cholangiocarcinoma; IO: immunotherapy; L: line; LN: lymph node; NHS: national health service; pCCA: perihilar cholangiocarcinoma; RT: radiotherapy; SACT: systemic anticancer treatment; IQR: interquartile range.

* For treatment management, values may not add to 100%, due to rounding.

** Targeted therapy was administered as single agent in the 2^nd^ or 3^rd^ line of the included patients; it was not given in combination with other SACT.

## Clinical implications and survival benefit

The ultimate clinical relevance of successful molecular profiling lies in its ability to guide treatment and improve patient outcomes. The patients’ therapeutic management is presented in Table 1. From the 97 (63%) fit for active palliative treatment, 1 (1%) female patient with initial Eastern Oncology Cooperative Group performance status of 0 received radiotherapy alone due to localized disease not amenable to surgery, rapid decline in her performance status and death before the chance for systemic treatment. This patient was excluded from the final survival analysis. With a median follow-up of 28.6 months, the mOS for patients receiving active palliative systemic treatment was 12.5 months, comparable to published real-world data and reflecting the recent gains from first-line ICI-chemotherapy combinations [4, 5, 18]. Patients referred for BSC had a mOS of only 5.2 months, reflecting the often-late presentation and rapid functional decline of patients with CCA. Notably, 37% of the total cohort were deemed suitable only for BSC, a finding that is consistent with other real-world series, in which 20–50% of patients are deemed unsuitable for systemic therapy owing to poor performance status or comorbidities [19]. The aggressive tumour biology, coupled with the often-silent clinical course leading to late diagnosis, limits the window for therapeutic intervention in many patients.

Crucially, the small subset of 9 patients who received targeted treatment with ivosidenib (*N* = 6, 4%) or pemigatinib (*N* = 3, 2%) demonstrated a statistically significant survival benefit compared to those receiving other active systemic treatments (*N* = 87, 56%). The mOS of this population was not reached (IQR: 23 months-NA) whereas for patients on other active systemic treatment it was 11.7 months (IQR: 6.8–20.5), Hazard Ratio 0.27; 95% CI 0.10–0.75, *p* = 0.012 (Supplemental Figure 3). This significant survival advantage achieved despite the retrospective design and small sample size, reinforces the clinical utility of a precision medicine approach in CCA.

## Limitations

While our results demonstrate the feasibility of genomic profiling in confirmed cases, we acknowledge that the requirement for pathological confirmation before treatment initiation may exclude a subset of patients who are too frail to undergo invasive sampling. Furthermore, as a referral centre, our data represents patients who successfully navigated the diagnostic pipeline; therefore, we cannot account for the total number of suspected cases where biopsy was deemed unfeasible or resulted in complications. These real-world barriers remain a significant factor in the implementation of precision oncology in advanced CCA.

## Conclusion and future directions

This study highlights that molecular profiling in advanced CCA is feasible in the majority of patients, with core biopsy techniques (EUS-FNB and percutaneous/surgical biopsy) demonstrating the highest success rates. It also provides real-world evidence confirming that the identification and subsequent treatment of actionable genomic alterations translate into a survival benefit.

The findings underscore several critical challenges and opportunities:

1. **Optimise Tissue Acquisition:** Preference should be given to methods that yield core biopsy material to ensure sufficient tumour cellularity for comprehensive genomic analysis. A statement on the tumour cellularity should be included within the pathology reports, as this information is critical for clinicians to assess the suitability of the tissue and the potential for successful molecular profiling. When possible, re-assessment of alternative tissue blocks and/or re-biopsy should be considered, where appropriate. However, we must acknowledge the inherent anatomical challenges in CCA, particularly in perihilar (pCCA) cases, where the infiltrative nature of the disease often makes obtaining high-quality diagnostic tissue exceptionally difficult.
2. **Expand Access to Testing:** Nearly 40% of tumours did not undergo molecular testing, primarily due to patient management with BSC or poor tissue quality. Future efforts must focus on integrating minimally invasive techniques (e.g. liquid biopsy) to enable earlier diagnosis/screening and to enhance profiling potential.
3. **Integrate Early Precision:** As trials such as SAFIR-ABC10 [20] investigate the role of targeted therapy as maintenance and other as first-line treatment [NCT 06282575, NCT 06501625], the need for successful molecular profiling upon diagnosis will become even more critical to maximise patient eligibility for these innovative strategies.

In an era of rapid therapeutic advancement for CCA, this study serves as a timely reminder: precision starts with a successful biopsy. Optimising the technical aspects of tissue acquisition is an essential, often overlooked, prerequisite to unlocking the full potential of personalised medicine and improving outcomes for patients with this aggressive malignancy.

## Supplementary Material


