# Supplementary material for: Tumour acquisition method and molecular profiling success in advanced cholangiocarcinoma
Source: Acta Oncol. 2026 May 7;65:45466. doi: 10.2340/ao.v65.45466 (PMC13162784; doi:10.2340/ao.v65.45466)
Supplement: Supplementary file 1 [file AO-65-45466-s1.pdf]

Supplementary material has been published as submitted. It has not been copyedited, or typeset by Acta Oncologica

Supplemental Table 1. Molecular profiling

Variable	Molecular profiling requests (N=98)	Molecular profiling success (N=87)	p value
Primary			p= 0.575
iCCA	73 (75%)	63/73 (86%)	
pCCA	13 (13%)	12/13 (92%)	
dCCA	12 (12%)	12/12 (100%)	
Tissue acquisition method			p=0.381
Surgical/Percutaneous biopsy	78 (80%)	70/78 (90%)	
EUS-FNB	5 (5%)	5/5 (100%)	
EUS-FNA	9 (9%)	7/9 (78%)	
Brushings	6 (6%)	5/6 (83%)	
Acquired tissue			p=0.645
Liver	71 (73%)	62/71 (87%)	
Bile duct	16 (16%)	15/16 (94%)	
LN	3 (3%)	3/3 (100%)	
Duodenum	1 (1%)	1/1 (100%)	
Omentum/peritoneum	4 (4%)	4/4 (100%)	
Bone	3 (3%)	2/3 (67%)	
Molecular testing assay			p=0.483
Foundation medicine	19 (19%)	16/19 (84%)	
NHS genomics	79 (81%)	71/79 (90%)	
Report of tumour cellularity (in samples requested for molecular profiling)	65 (66%)	60/65 (92%)	p=0.120
Adequacy of tumour cellularity*			p= 0.086
Yes	58 (59%)	55/58 (95%)	
No	7 (7%)	5/7 (71%)	

Values are n (%). Categorical variables were compared using the Chi-square test or the Fisher's exact test, as appropriate. A p value <0.05 was considered statistically significant.

** Adequacy of tumour cellularity has been previously defined as ≥20% tumour cellularity by Gambardella et al., 2021*

dCCA: distal cholangiocarcinoma, EUS-FNA: endoscopic ultrasound fine needle aspiration, EUS-FNB: endoscopic ultrasound fine needle biopsy, iCCA: intrahepatic cholangiocarcinoma, LN: lymph node, NHS: national health service, pCCA: perihilar cholangiocarcinoma

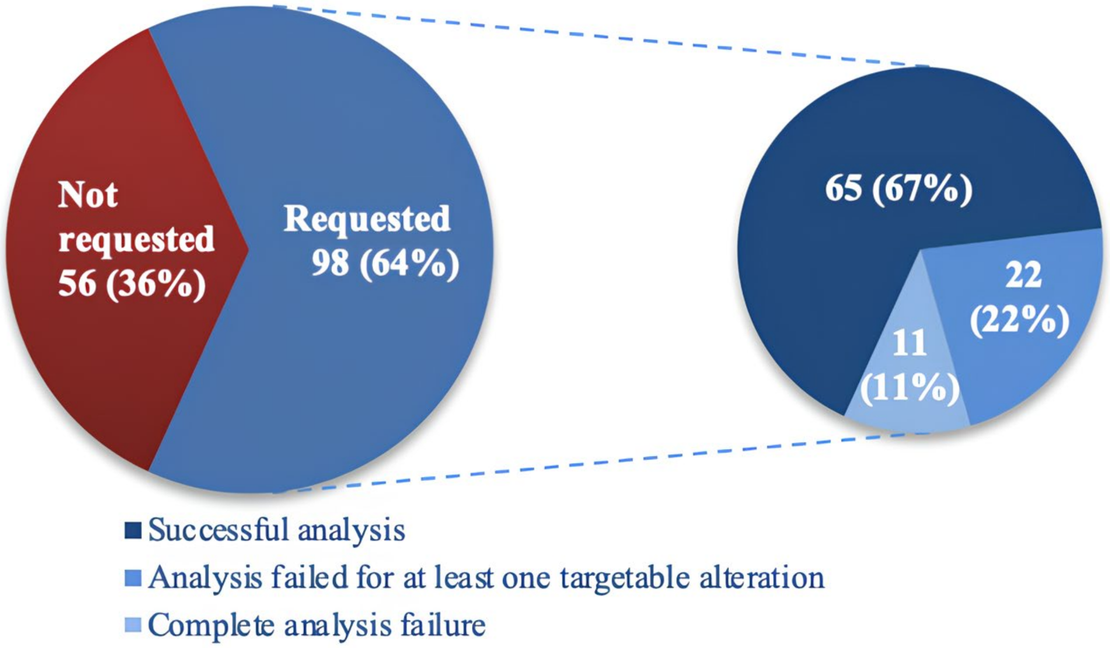

Supplemental Figure 1. Molecular profiling requests and success rate.

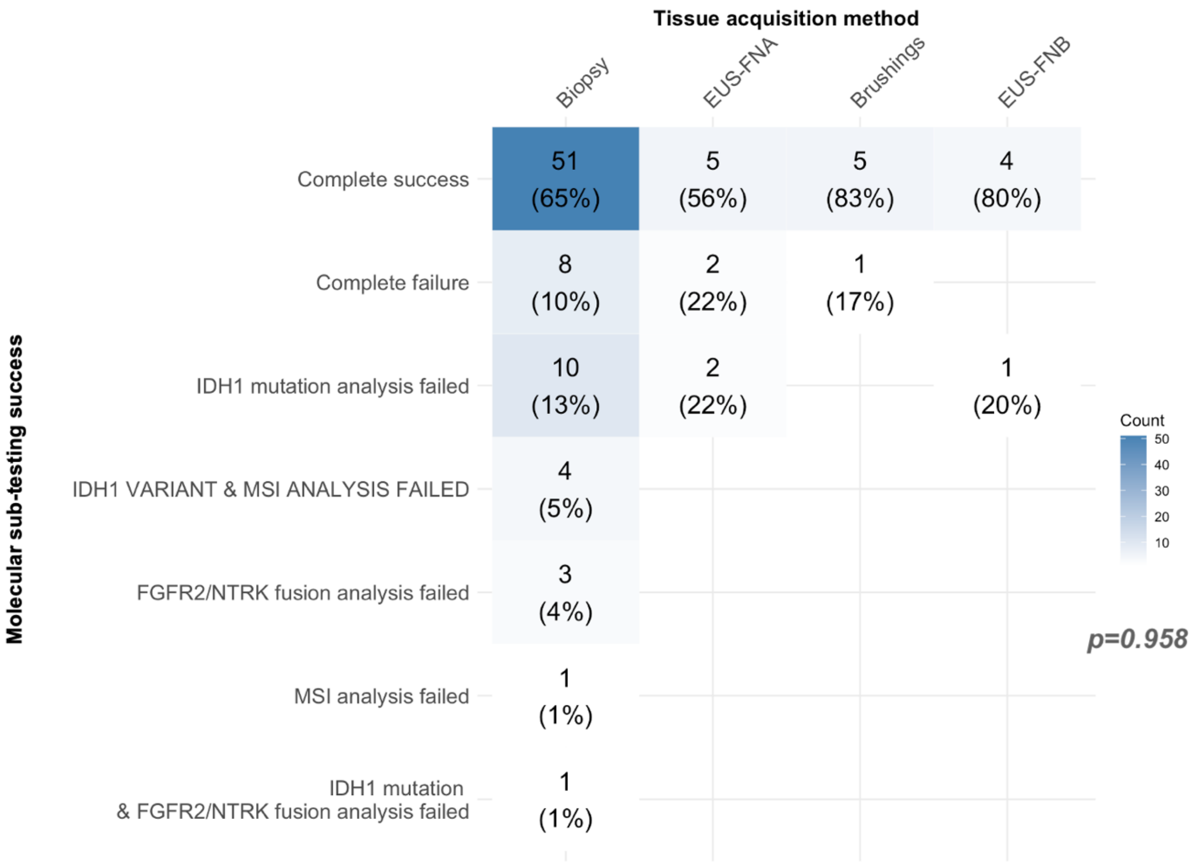

Supplemental Figure 2. Molecular sub-testing success based on tissue acquisition method.

Comparisons were made using the Fisher's exact test. A p value <0.05 was considered statistically significant.

EUS-FNA: endoscopic ultrasound-fine needle aspiration, EUS-FNB: endoscopic ultrasound-fine needle biopsy, FGFR2: fibroblast growth factor receptor 2, IDH1: isocitrate dehydrogenase 1, NTRK: neurotrophic tyrosine receptor kinase

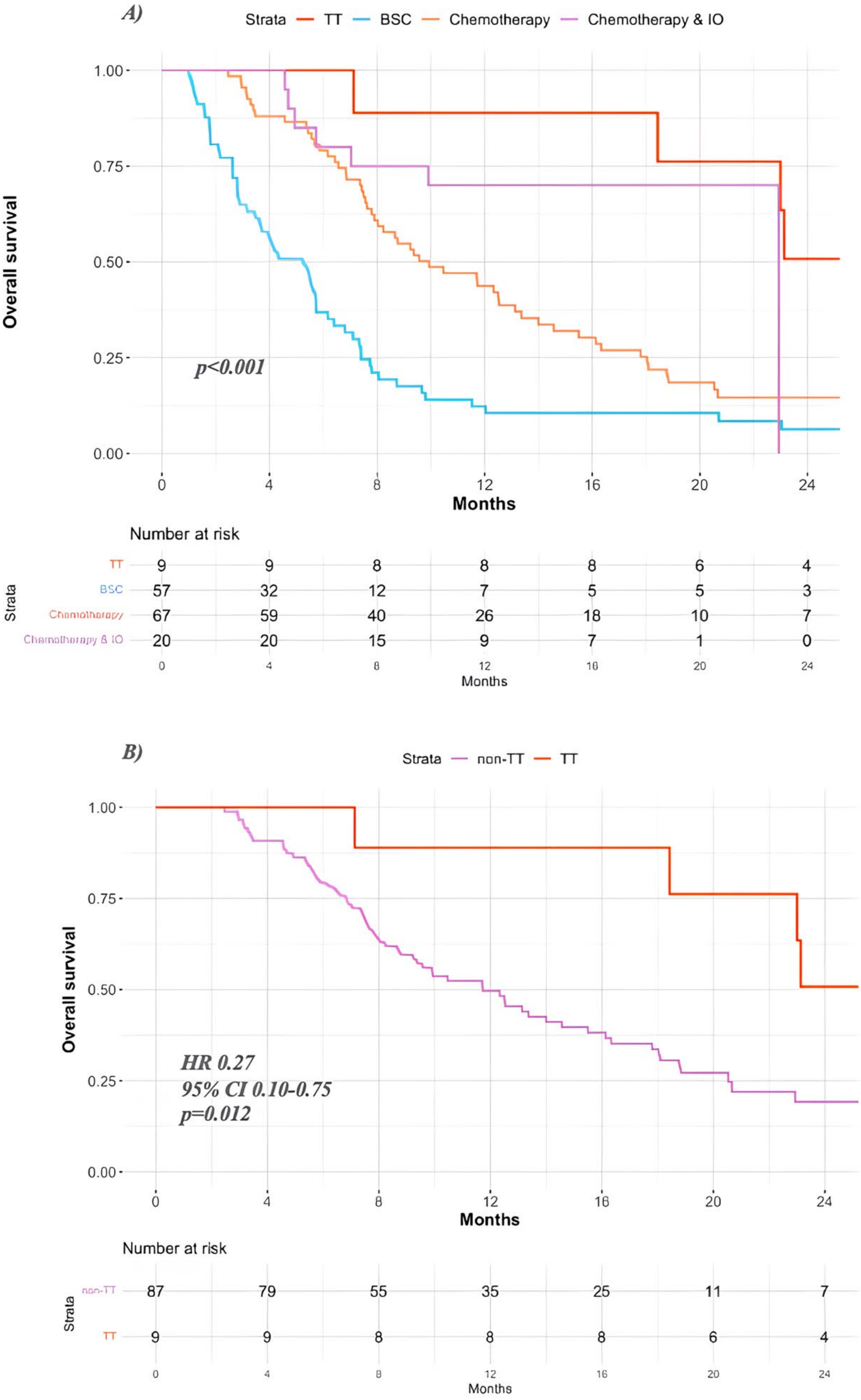
A)
Strata TT BSC Chemotherapy Chemotherapy & IO
Overall survival
1.00
0.75
0.50
0.25
0.00
p<0.001
0 4 8 12 16 20 24
Months
Number at risk
TT 9 9 8 8 8 6 4
BSC 57 32 12 7 5 5 3
Chemotherapy 67 59 40 26 18 10 7
Chemotherapy & IO 20 20 15 9 7 1 0
Strata
Months
B)
Strata non-TT TT
Overall survival
HR 0.27
95% CI 0.10-0.75
p=0.012
Months
Number at risk
non-TT 87 79 55 35 25 11 7
TT 9 9 8 8 8 6 4
Strata
Months

Supplemental Figure 3. A) Survival curves based on patient management. B) Survival curves between patients that received targeted treatment versus those on other active palliative systemic therapies.

Kaplan Meier and the log rank test were performed for survival analysis. A p value <0.05 was considered statistically significant.

BSC: best supportive care, IO: immunotherapy, TT: targeted therapy.
